# A Prospective Randomized Study Comparing the Bolus Doses of Norepinephrine and Phenylephrine for the Treatment of Spinal Induced Hypotension in Cesarean Section

**DOI:** 10.7759/cureus.27166

**Published:** 2022-07-23

**Authors:** J. P Tiwari, Sarv J Verma, Abhishek K Singh

**Affiliations:** 1 Department of Anaesthesia and Critical Care, Rajarshi Dashrath Autonomous State Medical College, Ayodhya, IND

**Keywords:** caesarean section, hypotension, phenylephrine, norepinephrine, epinephrine

## Abstract

Background: Spinal anesthetic-induced hypotension is the most worrisome complication for patients undergoing cesarean section under spinal anesthesia. The present study compares norepinephrine and phenylephrine bolus for the treatment of hypotension during spinal anesthesia for cesarean section.

Methods: One hundred twenty- six women aged between 22 and 40 years with singleton pregnancy classified to the American Society of Anesthesiologists (ASA) physical class I and II posted for elective cesarean section under spinal anesthesia were randomly divided into two groups of 63 each. Group I patients received phenylephrine 50 mcg (microgram) as an intravenous bolus, and Group II received 4 mcg of norepinephrine as an intravenous bolus to treat spinal hypotension.

Results: On comparing the demographic data of the patients in terms of age, weight, height, ASA Grade, level of block and surgery time no significant differences were found between the groups. Similarly, the fetal parameters were found to be not significantly different between the groups. However, the number of bolus doses of vasopressors required for the treatment of spinal-induced hypotension was significantly reduced in Group II (p=0.02). The frequency of bradycardia was found to be higher in patients who were given phenylephrine as compared to patients administered noradrenaline boluses (p=0.03). Five (7.93%) patients had shivering in Group I, while similar episodes were observed in 10 (15.87%) patients (p=0.05). Moreover, no significant difference was observed in comparing the heart rate and mean arterial pressure between the groups.

Conclusion: Intermittent boluses of norepinephrine are found to be effective in the management of spinal‑induced hypotension during caesarean section.

## Introduction

Cesarean sections are routinely performed in obstetrical procedures under spinal anesthesia to evade the danger of complications related to the airway and to prohibit the neonatal drug transfer linked with general anesthesia. Though the loading of fluid is maintained, the risk of maternal hypotension after spinal anesthesia cannot be avoided [1]. Common symptoms associated with hypotension are nausea, vomiting, dizziness, decreased uterine blood flow leading to fetal hypoxia, and acidosis. Management of hypotension comprises adequate use of vasopressors and intravenous fluids to inhibit neonatal and maternal effects. Vasopressors are highly useful in preventing and treating post‑spinal hypotension in parturients undergoing cesarean delivery [2].

Various modalities have been tried in the past to minimize the detrimental effects of spinal-induced hypotension. Non-pharmacological agents like application of tourniquet or leg wrappings along with pharmacological agents like small doses of local anesthetics, mixing adjuvants (fentanyl, sufentanil, dexmedetomidine, clonidine, etc.) with local anesthetics were used in the past, but no particular technique was found to be totally safe [2]. 

Among various vasopressors, phenylephrine (PE) is an alpha‑adrenergic receptor agonist widely recommended for the prevention/treatment of spinal‑induced hypotension [3]. In comparison with ephedrine, phenylephrine has better hemodynamic stability and causes minimal fetal hypoxia and acidosis, but it carries the risk of slowing maternal heart rate (bradycardia) and reduction in cardiac output (CO) progressively [4]. On the other hand, the literature reveals that the reflex slowing of heart rate (a surrogate marker of CO) may lead to compromise in uteroplacental perfusion, potentially adversely affecting a compromised fetus [5]. Norepinephrine (NE) is another alpha‑adrenergic receptor agonist with relatively weak agonistic activity at beta‑adrenergic receptors, which is considered to have the same effect as that of PE. There is a lower incidence of bradycardia with norepinephrine than with phenylephrine together, with a minimal decrease in cardiac output due to its mild beta‑agonist activity [6,7]. Considering this, we designed the present study to compare norepinephrine and phenylephrine bolus for the treatment of hypotension during spinal anesthesia for cesarean section.

## Materials and methods

The prospective randomized study was performed at a tertiary care hospital from March 2022 to June 2022 after permission from the Institutional Ethical Committee with letter number "Rajarshi Dashrath Autonomous State Medical College, Ayodhya, RD-ASMC/IEC/21-09" and due written consent from the patients. The parturients aging within 22- 40 years with singleton pregnancy classified to the American Society of Anesthesiologists (ASA) physical class I and II posted for elective cesarean section under spinal anesthesia were included in the study. Exclusion criteria include patients with hypersensitivity or allergic reaction to phenylephrine or norepinephrine, pregnancy-induced hypertensive (PIH), twins/multiple pregnancies, cardiovascular or cerebrovascular disease, and fetal abnormalities. 

A total of 63 patients were enrolled in the study, and all were randomized into two groups using the chit and box method. Group I patients received phenylephrine 50 mcg (microgram) as an intravenous bolus and Group II received 4 mcg of norepinephrine as an intravenous bolus to treat spinal-induced hypotension. All patients were premedicated with Oral Ranitidine 150 mg and Oral Metoclopramide 10 mg the night before the surgery. In the operation theatre, standard monitors were applied, and a 20-gauge intravenous cannula was inserted. Patients were preloaded with 10 ml/kg intravenous fluid (Ringer's lactate) before the administration of spinal anesthesia. 

Spinal anesthesia was administered in a sitting position at the L3-L4 position with all aseptic precautions. Injection Bupivacaine 0.5% (10 mg) together with 25 mcg of Inj Fentanyl was given in the intrathecal space. In the intra-operative period, parameters such as maternal heart rate and mean arterial pressure, number of intravenous bolus doses of norepinephrine or phenylephrine, the frequency of bradycardia, shivering, nausea/vomiting in the mother, and fetal outcomes such as Apgar score and umbilical vein blood gases, etc., were recorded.

Statistical analysis

All the results of the study were compiled and entered into a Microsoft Excel (Microsoft® Corp., Redmond, WA) sheet. The data analysis was done using IBM Corp. Released 2012. IBM SPSS Statistics for Windows, Version 21.0. Armonk, NY: IBM Corp. All parametric data were analyzed by "Student's t-test", while non-parametric data were analyzed by the Chi-Square test. p<0.05 was taken as statistically significant.

## Results

All patients were successfully enrolled and completed the study. The mean age (years) was 29.4 ± 4.82 in Group I and 30.2 ± 5.09 in Group II (p=0.73), mean weight (kilograms) was 75.2 ± 8.89 and 78.4 ± 9.14 in Group I & II, respectively (p=0.89), and height (centimeters) was 155.6 ± 7.82 in Group I and 157.2 ± 8.69 in Group II (p=0.28) (Table 1). On comparing the level of dermatomal block, statistically insignificant results were obtained between the groups (p=0.67). Similar, statistically insignificant results were obtained in comparing both the groups for surgical time (duration of surgery, induction to delivery, skin incision to delivery, and uterine incision to delivery) (Table 1).

**Table 1:** Demographic data of the patients T3: Third thoracic vertebra level; T4: Fourth thoracic vertebra level; T5: Fifth thoracic vertebra level.

Parameters		Variables	Group I	Group II	P value
Age (years)			29.4 ± 4.82	30.2 ± 5.09	0.73
Weight (kilograms)			75.2 ± 8.89	78.4 ± 9.14	0.89
Height (centimeters)			155.6 ± 7.82	157.2 ± 8.69	0.28
ASA	I/II		38/25	40/23	-
Level of dermatomal block	T3		2	1	0.67
	T4		48	50	
	T5		13	12	
Surgical time (minutes)	Duration of surgery		68.3 ± 6.61	70.4 ± 5.73	0.34
	Induction to delivery		10.6 ± 1.09	11.2 ± 1.23	0.09
	Skin incision to delivery		5.9 ± 0.72	5.3 ± 0.98	0.81
	Uterine incision to delivery		2.14 ± 0.23	1.91 ± 0.59	0.29

In Group I and Group II, the mean PO2 (partial pressure of oxygen) (mm Hg) was 28.3 ± 2.23 and 25.4 ± 2.89 (p=0.08), mean PCO2 (partial pressure of carbon dioxide ) (mm Hg) was 42.8 ± 3.61 and 46.5 ± 2.51 (p=0.12), umbilical pH was 7.32 ± 0.93 and 7.39 ± 1.19, Apgar score at 1 minute was 8.01 ± 1.73 and 7.92 ± 1.98 (p=0.26), Apgar score at 5 minutes was 8.91 ± 1.68 and 8.87 ± 1.43 (p=0.39) and lactate levels was 1.93 ± 0.48 and 2.21 ± 0.17 (p=0.19), respectively (Table 2).

**Table 2:** Comparison of fetal parameters PO2: Partial pressure of oxygen; PCO2: Partial pressure of carbon dioxide

Variables	Group I	Group II	P value
PO2 (mm Hg)	28.3 ± 2.23	25.4 ± 2.89	0.08
PCO2 (mm Hg)	42.8 ± 3.61	46.5 ± 2.51	0.12
Umbilical ph	7.32 ± 0.93	7.39 ± 1.19	0.07
Apgar at 1 minute	8.01 ± 1.73	7.92 ± 1.98	0.26
Apgar at 5minutes	8.91 ± 1.68	8.87 ± 1.43	0.39
Lactate	1.93 ± 0.48	2.21 ± 0.17	0.19

The number of bolus doses of vasopressors required for the treatment of spinal-induced hypotension was significantly reduced in Group II (p=0.02) (Table 3). The frequency of bradycardia was found to be higher in patients who were given phenylephrine as compared to patients administered noradrenaline boluses (p=0.03). Five (7.93%) patients had shivering in Group I, while similar episodes were observed in 10 (15.87%) patients (p=0.05) (Table 3).

**Table 3:** Number of doses of vasopressors with frequency of bradycardia, nausea/vomiting and shivering

Variables	Group I	Group II	P value
Number of boluses of vasopressors	2.36 ± 1.89	1.42 ± 1.12	0.02*
Bradycardia	12 (19.04%)	3 (4.76%)	0.03*
Nausea/vomiting	5 (7.93%)	5 (7.93%)	-
Shivering	5 (7.93%)	10 (15.87%)	0.05*

The mean heart rate (beats/min) in Groups I and II at baseline was 96.2 ± 5.59 and 96.2 ± 5.71 (p=0.89), respectively. On comparing the mean heart rate at two, four, six, eight, 10, 15, 20, 25 & 30 minutes, the results were obtained as statistically insignificant (Table 4). 

**Table 4:** Comparison of heart rate (beats/min)

Time (minutes)	Group I	Group II	P value
0	96.2 ± 5.59	96.2 ± 5.71	0.89
2	90.4 ± 4.89	96.0 ± 4.53	0.24
4	88.2 ± 3.79	94.2 ± 3.82	0.37
6	84.6 ± 4.41	94.0 ± 5.37	0.07
8	82.1 ± 4.91	92.6 ± 5.19	0.07
10	80.2 ± 3.51	90.4 ± 4.92	0.06
15	79.2 ± 4.46	88.4 ± 5.03	0.10
20	78.8 ± 3.97	86.2 ± 4.42	0.29
25	78.0 ± 4.61	85.4 ± 3.89	0.41
30	76.4 ± 3.09	80.6 ± 4.12	0.71

The mean arterial pressure (mm Hg) in Groups I and II at baseline was 77.8 ± 4.69 and 76.9 ± 5.01 (p=0.89), respectively. On comparing the mean arterial pressure at two, four, six, eight, 10, 15, 20, 25 & 30 minutes, the results were obtained as statistically insignificant (Table 5). 

**Table 5:** Comparison of mean arterial pressure (mm Hg)

Time (minutes)	Group I	Group II	P value
0	77.8 ± 4.69	76.9 ± 5.01	0.89
2	76.9 ± 4.93	76.4 ± 4.31	0.91
4	74.1 ± 5.22	74.8 ± 5.77	0.79
6	73.5 ± 5.72	73.0 ± 4.83	0.81
8	72.7 ± 5.41	73.1 ± 5.47	0.83
10	73.8 ± 4.89	74.0 ± 4.72	0.65
15	72.3 ± 5.83	72.6 ± 4.48	0.32
20	71.4 ± 4.65	71.8 ±4.31	0.53
25	70.9 ±3.96	71.2 ± 4.11	0.61
30	70.3 ± 4.68	70.1 ± 4.76	0.94

## Discussion

Spinal hypotension is a common complication resulting in women undergoing cesarean section. In such patients, to avoid it, phenylephrine is considered a fairly better option [8]. Vasopressors as an infusion or as intermittent boluses may help treat spinal hypotension [9]. It has the advantage that there is a minimum requirement of anesthetist intervention during cesarean sections [10]. The intermittent boluses can be practical in poor-resource settings where infusion pumps are not available or are inadequate and therefore cannot be available to all parturients who undergo a cesarean section [11]. Norepinephrine infusion may be regarded alternative to phenylephrine to treat spinal-induced hypotension for cesarean delivery. It could be more advantageous than phenylephrine as it causes less reduction in heart rate and cardiac output [12,13]. This study compared norepinephrine and phenylephrine bolus for the treatment of hypotension during spinal anesthesia for cesarean section.

Our results showed that the mean age (years) was 29.4 ± 4.82 and 30.2 ± 5.09, weight (kilograms) was 75.2 ± 8.89 and 78.4 ± 9.14, height (centimeter) was 155.6 ± 7.82 and 157.2 ± 8.69, ASA grade I was seen in 38 and 40 patients and II in 25 and 23 patients. Dermatomal block T3 was seen in two and one patients, T4 in 48 and 50 patients and T5 in 13 and 12 patients. Duration of surgery (minutes) was 68.3 ± 6.61 and 70.4 ± 5.73, induction to delivery was 10.6 ± 1.09 and 11.2 ± 1.23, skin incision to delivery was 5.9 ± 0.72 and 5.3 ± 0.98, and uterine incision to delivery was 2.14 ± 0.23 and 1.91 ± 0.59 in Group I and Group II, respectively. Puthenveettil et al. conducted a study on 50 patients undergoing elective cesarean section under spinal anesthesia [12]. In this prospective observational study, the researchers compared the effectiveness of bolus doses of phenylephrine were compared with noradrenaline to treat spinal-induced hypotension. This study consists of two groups of 25 patients each. The authors administered 50 mcg of phenylephrine in one group and 4 mcg of noradrenaline as an intravenous bolus to treat spinal-induced hypotension in another group. In the study, the number of bolus doses of vasopressor agents required to treat spinal-induced hypotension was significantly lower in group N as compared to group P (1.40 ± 0.577 vs. 2.28 ± 1.061, P = 0.001) [12]. In the phenylephrine group, the frequency of bradycardia was high as compared to the noradrenaline group, although this difference was statistically insignificant (P = 0.192). The fetal parameters (Umbilical cord blood analysis and APGAR score) were comparable between the two groups [12]. Maternal complications such as nausea/vomiting and shivering were also insignificant between the noradrenaline and phenylephrine groups [12].

Our results showed that in Groups I and II, the mean PO2 was 28.3 ± 2.23 and 25.4 ± 2.89, PCO2 was 42.8 ± 3.61 and 46.5 ± 2.51, umbilical pH was 7.32 ± 0.93 and 7.39 ± 1.19, Apgar score at 1 minute was 8.01 ± 1.73 and 7.92 ± 1.98, Apgar score at 5 minutes was 8.91 ± 1.68 and 8.87 ± 1.43 and lactate was 1.93 ± 0.48 and 2.21 ± 0.17, respectively (p<0.05). Goel et al., in their study on 200 patients, determined the effect of phenylephrine and norepinephrine infusion. Parameters such as intraoperative nausea vomiting (IONV), maternal systolic blood pressure (SBP), heart rate (HR), and fatal Apgar scores were assessed and compared [13]. Patients were divided into two groups of 100 each. Two groups, A and B, received phenylephrine and norepinephrine, respectively, targeting maintenance of SBP to 100% of the baseline value. Results showed that there was statistically significantly lower SBP during the first six minutes following intrathecal injection in group A. Group A (13%) showed more parturients experienced ≥1 episode of hypotension than Group B (9%). There was a 16% incidence of bradycardia in group A and 1% in group B, which was statistically significant (P< 0.05) [13]. The episodes of hypertension, IONV, maternal vasopressor consumption, and neonatal Apgar score were comparable among both groups [13].

Our results showed that the number of boluses of vasopressors requirement was 2.36 ± 1.89 in Group I and 1.42 ± 1.12 in Group II. Bradycardia was seen among 12 (19.04%) in Group I and three (4.76%) in Group II, nausea/vomiting seen in five (7.93%) in Group I and five (7.93%) in Group II, and shivering in five (7.93%) Group I and 10 (15.87%) Group II. Our results showed a statistically insignificant difference in heart rate and mean arterial pressure in both groups. Mon et al. studied cardiac output changes with phenylephrine and ephedrine and observed that even though cardiac output and HR were maintained better with ephedrine, less neonatal acidosis was noted with the use of phenylephrine [14].

Limitations

First, a large sample size may reveal more extensive results and can give a better idea of fetal and maternal side effects. Second, the study mentions the monitoring of mean arterial pressure without assessment of cardiac output, which can be a better predictor of maternal hemodynamics. 

## Conclusions

The study advocates the use of intermittent boluses of norepinephrine in the effective management of spinal‑induced hypotension during cesarean section. Although the hemodynamic variables are stable with the usage of intravenous boluses of noradrenaline and phenylephrine, the number of doses of vasopressor use was found to be significantly more with the use of phenylephrine. In the noradrenaline group, the episodes of bradycardia are significantly less as compared to the phenylephrine group. On the contrary, in the parturients where phenylephrine was administered as an intravenous bolus dose, shivering was observed to be significantly less as compared to noradrenaline use in other groups. 
